# 系统性肥大细胞增生症11例回顾性分析

**DOI:** 10.3760/cma.j.issn.0253-2727.2023.04.013

**Published:** 2023-04

**Authors:** 龙 常, 路 张, 炎 张, 俊 冯, 道斌 周, 欣欣 曹

**Affiliations:** 中国医学科学院、北京协和医学院北京协和医院血液内科 100730 Department of Hematology Peking Union Medical College Hospital, Chinese Academy of Medical Sciences & Peking Union Medical College, Beijing 100730, China

肥大细胞增生症是由异常肥大细胞克隆性增殖并在一个或多个器官系统中聚集导致的一组异质性疾病。在2017年世界卫生组织（WHO）分类中，被单独列为一种具有独立临床和病理特征的血液肿瘤[1]。根据病理特征、发病部位和临床表现分为三大亚类：皮肤型肥大细胞增多症（cutaneous mastocytosis，CM）、系统性肥大细胞增生症（systematic mastocytosis，SM）和肥大细胞肉瘤。SM临床中较罕见，截至2020年，欧洲肥大细胞增生症研究网络（ECNM）共登记注册了2 350例肥大细胞增生症患者，其中SM患者1 713例[2]。自1980年冯耀庭等[3]在国内首次报道SM以来，迄今全国仅有20余例个案报道，临床资料较少，无成组队列研究。SM临床表现异质性较大，受累脏器较多，诊断相对复杂，为提高临床医师对于此病的认识，本文将我院确诊的11例SM患者进行总结。

## 病例与方法

1. 病例：本研究回顾性分析1992年1月1日至2021年12月31日就诊于北京协和医院并确诊为SM的患者。所有患者符合WHO 2017分类中SM的诊断标准。评估项目包括患者人口学资料、临床表现、血液检查、骨髓涂片和活检、骨髓流式细胞术、基因检测、受累脏器病理检测等相关实验室检查结果及治疗。

2. 疗效评估：疗效评估标准参照2013年IMG-ECNM侵袭性SM（ASM）的疗效评估标准，分为完全缓解（CR）、部分缓解（PR）、临床改善（CI）、疾病稳定（SD）和疾病进展（PD）。总生存（OS）时间定义为自SM诊断至患者死亡或末次随访的时间间隔。无进展生存（PFS）时间定义为自SM诊断至任何原因导致疾病进展、死亡或末次随访的时间间隔。

3. 随访：通过查阅患者门诊、住院病历及电话询问等方式完成随访。随访截止时间为2022年4月30日。

4. 统计学处理：用Graphpad Prism 8.0.2软件进行数据分析，连续变量采用中位数（范围）表示，计数资料用例数表示。生存曲线采用Kaplan-Meier法绘制，OS和PFS采用Kaplan-Meier法进行分析。

## 结果

1. 临床特征和实验室检查：患者临床特征见表1。临床表现方面，皮肤潮红5例，发热4例，意识丧失、低血压、皮疹、腹胀各3例，水肿、腹泻、呕吐、呼吸困难各2例，乏力、关节痛、无症状各1例。9例患者行骨髓流式细胞术和基因检测。8例表达CD117，5例表达CD25，5例表达CD2，4例表达CD33，2例表达CD4，2例表达CD38。3例［2例ASM，1例SM伴血液系统肿瘤（SM-AHN）］患者KIT D816V突变阳性，其中1例合并SRSF2和IDH2突变，1例合并SRSF2和TET2突变。1例肥大细胞白血病（MCL）患者KIT K509I突变阳性（后证明为胚系突变），5例未检测到突变。11例患者中仅1例ASM患者有基线和发作期的类胰蛋白酶水平，分别为25 ng/ml和82 ng/ml。

**表1:** 11例系统性肥大细胞增生症患者的临床特征、疗效和生存

例号	性别	年龄（岁）	起病至诊断间隔（月）	诊断分类	髓外受累脏器	MARS评分	肝脾肿大	骨髓MC比例（%）	基因测序	治疗方案	最佳疗效	生存状态	OS（月）
1	男	58	144	ISM	无	无	无	0.5	阴性	观察	SD	生存	105
2	男	17	1	ASM	脾、肝脏、骨、胸膜、腹膜、胃肠道	0	肝脾大	1.0	阴性	干扰素+糖皮质激素	PR	生存	70
3	女	27	3	MCL	子宫、皮肤	0	无	23.5	KIT K509I	伊马替尼	CR	生存	55
4	男	58	13	ASM	脾、肝脏、胸膜、淋巴结	1	肝脾大	2.0	KIT D618V、SRSF2 P95L、TET2 E1106RFs	干扰素	PD	死亡	7
5	男	42	6	ASM	脾、肝脏、胸膜、胃肠道	1	肝脾大	1.0	KIT D816V、SRSF2 C284A、IDH2 G419A	达沙替尼+糖皮质激素	PD	生存	34
6	男	34	126	SM-AHN	皮肤、淋巴结	0	肝大	0.5	KIT D618V	达沙替尼+DA	CR	生存	47
7	男	51	1	MCL	脾、肝脏、胸膜、腹膜	2	脾大	28.5	阴性	糖皮质激素	PD	死亡	3
8	女	28	38	MCL	脾、腹膜	0	脾大	30.0	阴性	伊马替尼	CR	生存	11
9	女	41	12	ASM	骨	0	无	2.0	阴性	干扰素+糖皮质激素	CR	生存	132
10	女	60	25	ASM	胃肠道	2	无	6.0	未查	姑息治疗	PD	死亡	1
11	男	72	0	ISM	胃肠道	无	无	1.0	未查	观察	SD	生存	145

**注** MC：肥大细胞；MCL：肥大细胞白血病；SM-AHN：系统性肥大细胞增生症伴血液系统肿瘤；ASM：侵袭性系统性肥大细胞增生症；ISM：惰性系统性肥大细胞增生症；MARS：突变调整的危险度积分；SD：疾病稳定；CR：完全缓解；PR：部分缓解；PD：疾病进展；DA：柔红霉素+阿糖胞苷；OS：总生存时间

2. 治疗和随访：患者的治疗方案及疗效评估见表1，随访至2022年4月30日，无失访病例，中位随访时间47（0～145）个月，死亡3例，均死于病情进展。中位OS、PFS时间均未达到（图1），不同类型的SM中位OS时间未达到（图2）。对9例进展期SM（AdvSM）进行危险分层，7例低危SM中位OS时间未达到，2例中危SM中位OS时间2个月，差异有统计学意义（*P*＝0.002）。

**图1:**
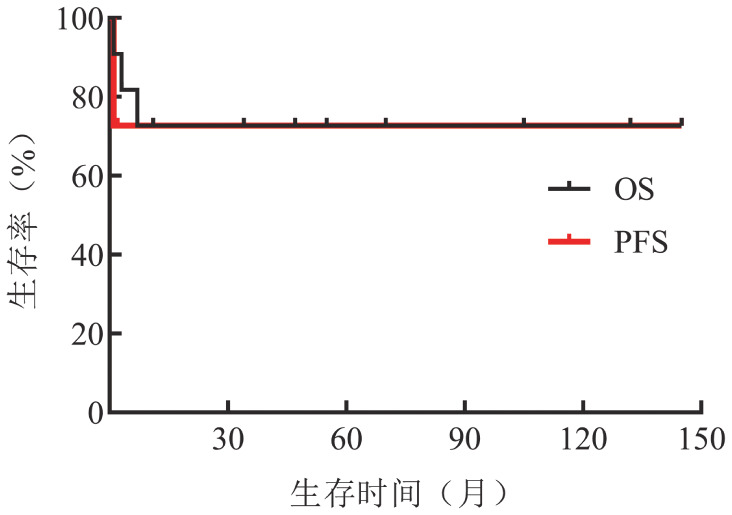
11例系统性肥大细胞增生症患者的总生存（OS）和无进展生存（PFS）曲线

**图2:**
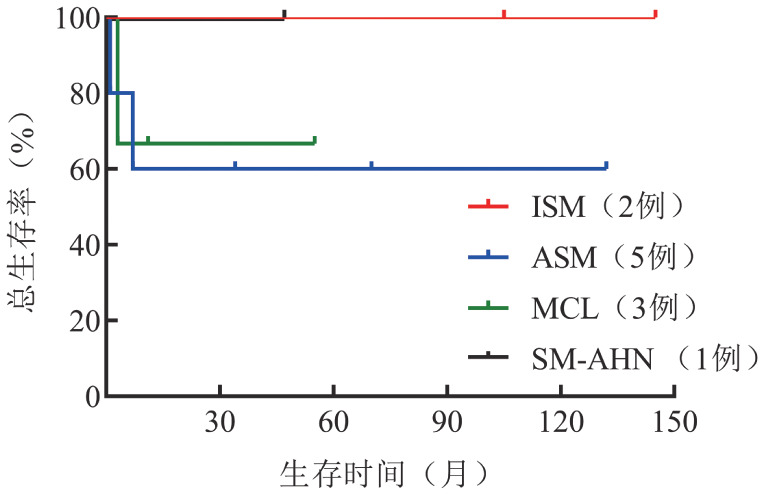
不同类型系统性肥大细胞增生症患者的总生存曲线 ISM：惰性系统性肥大细胞增生症；ASM：侵袭性系统性肥大细胞增生症；MCL：肥大细胞白血病；SM-AHN：系统性肥大细胞增生症伴血液系统肿瘤

## 讨论

SM是侵袭性肥大细胞在真皮组织以外的一个或多个系统发生克隆性增殖并释放大量活性介质，引起多器官功能障碍的一类疾病。根据2017年WHO分类标准，SM分为惰性SM（ISM）、冒烟性SM（SSM）、SM-AHN、ASM、MCL，后三者统称为AdvSM。成人患者常见于骨髓、皮肤、脾脏、淋巴结、肝脏和（或）单核-巨噬系统，约占肥大细胞增生症的10％[4]。SM临床表现多样，易造成诊断延迟和漏诊。2014年丹麦的一项回顾性研究结果显示SM的年发病率为0.89/10万，其中ISM比例最高，占82.5％。在ECNM登记注册的1 713例SM患者中，ISM占78.5％，ASM和SM-AHN分别占4.6％和12.1％[5]。与国外数据相比，国内报道病例以AdvSM居多，ISM和SSM相对罕见，推测前者因病情危重、进展迅速、血液系统受累严重而得到诊治。而后者常因病情较轻、无明显血液系统受累和脏器功能损伤、临床医师认识不足、缺乏相应的检查手段而漏诊。

临床表现方面，SM可以有全身症状、皮肤症状、炎症介质释放相关症状和肌肉骨骼症状（骨痛、肌痛、骨折）。梅奥诊所2010年回顾了342例SM患者，中位年龄49岁，以色素性荨麻疹、炎症介质释放症状和胃肠道症状多见[6]。本文11例患者中，常见的临床表现有发热、皮肤潮红、晕厥、呕吐、腹泻等，与文献报道一致。实验室诊断方面，血清类胰蛋白酶在肥大细胞脱颗粒过程中大量释放，对于诊断和鉴别诊断有重要作用，但在国内开展较少[7]。SM骨髓检查可见异常的肥大细胞，形态特点为梭形和幼稚形。流式细胞术中，CD117、CD25和CD2为最常见的免疫表型，也常有CD13、CD33、CD203c等的表达[8]。本文9例患者骨髓流式细胞术结果显示8例CD117阳性，5例CD25阳性，4例CD2阳性，其他常见的免疫表型还包括CD4、CD33、CD38等。SM患者中超过80％可检测到17号外显子KIT 816位点突变，随着病情的进展，AdvSM还会出现其他造血细胞髓系突变[9]。目前有证据表明SRSF2、ASXL1、RUNX1与预后不良相关，因此在突变调整的危险度积分中作为危险因素[10]。本文9例AdvSM中，8例完成基因测序，其中3例患者有KIT 816位点突变，发生比例较低，可能与本文中所有患者均为中低危有关。另外，3例患者为一代测序技术检测，敏感性较低。

不同类型的SM预后差别较大，ISM预期寿命接近正常；SSM中位OS时间约120个月；ASM预后较差，中位OS时间为41个月；MCL预后最差，OS时间仅为2个月；SM-AHN的预后取决于伴发的血液系统肿瘤，中位OS时间为24个月[11]。本文11例SM患者的中位OS和PFS时间均未达到，预后较既往报道好，可能与纳入较多低危患者相关。2例中危SM患者中位OS时间仅为2个月，与低危患者相比差异有统计学意义。

SM的治疗包括三个方面：针对肥大细胞的治疗如羟基脲、干扰素、传统化疗药物、酪氨酸激酶抑制剂（TKI）、异基因造血干细胞移植；针对肥大细胞炎症介质释放的治疗：白三烯抑制剂、H1/2受体拮抗剂、肾上腺素、糖皮质激素；针对器官损伤的治疗如抗骨质疏松、改善胃肠功能[12]。干扰素、羟基脲可降低肿瘤负荷，改善炎症介质释放症状，总有效率（ORR）为10％～40％，疗效维持时间约12个月[13]，不良反应较轻，且费用较低，本文中4例ASM患者接受干扰素治疗，ORR 75％，目前均长期维持。克拉屈滨是治疗SM的传统化疗药物，但增加骨髓抑制和感染的风险，没有获得FDA批准用于治疗SM，目前应用较少。第一代TKI伊马替尼治疗KIT D816V阳性患者的ORR仅为17％，治疗KIT突变阴性患者ORR为33％，目前用于治疗KIT突变阴性或突变情况不明的SM患者[14]。米哚妥林是多重酪氨酸激酶受体的小分子抑制剂，于2016年获批，治疗AdvSM的ORR为60％，且疗效与KIT突变类型无关[15]–[16]。阿伐替尼（Avapritinib）是针对KIT-D816V突变的高效、高选择性抑制剂，治疗AdvSM患者的ORR为77％，90％以上患者骨髓肥大细胞数量、类胰蛋白酶水平和KITD816V突变负荷明显下降，80％患者脾脏缩小，中位生存期未达到。DCC-2618是一种口服生物利用、选择性的KIT和PDGFRA的开关控制抑制剂，针对KIT和PDGFRA的野生型和突变型，在其开关口袋结合位点与之结合，从而防止这些激酶从无活性构象转变为有活性构象，临床研究正在进行中[17]。米哚妥林和阿伐替尼价格昂贵。本文4例患者接受TKI治疗，2例KIT D816V阴性患者接受伊马替尼治疗，1例CR，1例PR。2例KIT D816V阳性患者接受达沙替尼治疗，1例CR，1例PD。传统TKI仍然是国内临床中最常见的针对肥大细胞的治疗手段。异基因造血干细胞移植治疗SM数据较少，2014年一项国际多中心回顾性研究共纳入57例接受异基因造血干细胞移植的SM患者，3年OS率为57％，其中SM-AHN 38例，MCL 12例，ASM 7例，3年OS率分别为74％、43％、17％[18]。

总之，SM是临床上一类罕见疾病，临床异质性较大，国内医师对此病认识较少，需要提高认识，尽早诊断。对于临床表现为色素荨麻疹、介质释放症状的患者需要警惕SM，及时完善骨髓及受累脏器活检，病理检查是SM诊断的金标准。诊断后应进行临床分型，完善血清类胰蛋白酶，CD117、CD2、CD25组织化学染色，基因检测等以指导治疗。
